# Biological Markers of Cognitive Dysfunction In Bipolar Disorder Type 1: A Systematic Review And Meta-Analysis

**DOI:** 10.1192/j.eurpsy.2025.1064

**Published:** 2025-08-26

**Authors:** A. Melillo, C. Recano, M. Allegretta, A. Ambroselli, E. Caporusso, L. Giuliani, A. Perrottelli, P. Pezzella, G. M. Giordano, P. Bucci, A. Mucci, S. Galderisi

**Affiliations:** 1 Deparment of Physical and Mental Health and Preventive Medicine, University of Campania “L. Vanvitelli”, Naples, Italy

## Abstract

**Introduction:**

Bipolar disorder (BD) has a lifetime prevalence of approximately 2% and is one of the 17 most disabling diseases worldwide. Multiple factors, beyond the impact of acute mood episodes, contribute to the burden of the disease. BD is also characterized by cognitive impairment (CI), which affects between 30% and 57% of patients during euthymic phases [1]. The most impacted cognitive domains are memory, attention, processing speed, and executive function [1]. CI plays a critical role in the diminished psychosocial functioning observed in BD patients [2, 3]. However, cognitive profiles in BD are heterogeneous, and the underlying causes of CI are likely multifactorial, involving more than just the effects of acute mood episodes.

In recent years, growing evidence on the biomarkers associated with BD has expanded our understanding of various molecular pathways—such as inflammatory, oxidative stress, and dysmetabolic pathways—which have been linked to CI. These pathways not only affect cognitive function but also contribute to an increased risk of metabolic and inflammatory comorbidities, further exacerbating CI and the overall burden of the illness [4, 5]. As a result, further research into biomarkers of CI is essential, as it may enhance our knowledge of the molecular mechanisms involved and help identify potential pharmaceutical targets.

Despite recent systematic reviews on the topic, most have focused on individual pathways [2, 6], and there is a need for a more comprehensive review of the current evidence regarding biomarkers linked to CI in BD.

**Objectives:**

The goal of the present review is to examine the relationship between peripheral biomarkers (e.g., blood, saliva, urine) and impairments across specific cognitive domains in individuals with BD in euthymic state.

**Methods:**

Inclusion/exclusion criteria: we included studies that assessed patients in euthymic states at the time of both cognitive evaluation and biomarker collection, and we excluded studies employing more invasive techniques (e.g., spinal puncture). Study quality will be assessed using the Joanna Briggs Institute Critical Appraisal tools.

**Results:**

A database search (PubMed, Scopus, PsychInfo) identified 6,767 records after duplicate removal. Following title, abstract, and full-text screening, as well as manual reference list checks, a total of 52 studies were included. Data extraction is currently ongoing and includes publication year, country of origin, study design, sample size, sociodemographic and clinical characteristics, cognitive assessment tools, biomarkers investigated, and study outcomes.

**Conclusions:**

Conclusions will be drawn at the end of the systematic review process. Further research into biomarkers of CI in BD is essential, as it may enhance our knowledge of the molecular mechanisms involved and help identify potential pharmaceutical targets.

**Disclosure of Interest:**

None Declared

